# Characterizing Use of a Multicomponent Digital Intervention to Predict Treatment Outcomes in First-Episode Psychosis: Cluster Analysis

**DOI:** 10.2196/29211

**Published:** 2022-04-07

**Authors:** Shaunagh O'Sullivan, Lianne Schmaal, Simon D'Alfonso, Yara Jo Toenders, Lee Valentine, Carla McEnery, Sarah Bendall, Barnaby Nelson, John F Gleeson, Mario Alvarez-Jimenez

**Affiliations:** 1 Orygen Parkville Australia; 2 Centre for Youth Mental Health University of Melbourne Melbourne Australia; 3 School of Computing and Information Systems University of Melbourne Melbourne Australia; 4 Health Brain and Mind Research Centre Australian Catholic University Melbourne Australia; 5 School of Behavioural and Health Sciences Australian Catholic University Melbourne Australia

**Keywords:** digital intervention, digital health, youth mental health, psychotic disorders, clustering, usage metrics, log data, social networking

## Abstract

**Background:**

Multicomponent digital interventions offer the potential for tailored and flexible interventions that aim to address high attrition rates and increase engagement, an area of concern in digital mental health. However, increased flexibility in use makes it difficult to determine which components lead to improved treatment outcomes.

**Objective:**

This study aims to identify user profiles on Horyzons, an 18-month digital relapse prevention intervention for first-episode psychosis that incorporates therapeutic content and social networking, along with clinical, vocational, and peer support, and to examine the predictive value of these user profiles for treatment outcomes. A secondary objective is to compare each user profile with young people receiving treatment as usual (TAU).

**Methods:**

Participants comprised 82 young people (aged 16-27 years) with access to Horyzons and 84 receiving TAU, recovering from first-episode psychosis. In addition, 6-month use data from the therapy and social networking components of Horyzons were used as features for *K*-means clustering for joint trajectories to identify user profiles. Social functioning, psychotic symptoms, depression, and anxiety were assessed at baseline and 6-month follow-up. General linear mixed models were used to examine the predictive value of user profiles for treatment outcomes and between each user profile with TAU.

**Results:**

A total of 3 user profiles were identified based on the following system use metrics: low use, maintained use of social components, and maintained use of both therapy and social components. The *maintained therapy and social* group showed improvements in social functioning (*F*_2,51_=3.58; *P*=.04), negative symptoms (*F*_2,51_=4.45; *P*=.02), and overall psychiatric symptom severity (*F*_2,50_=3.23; *P*=.048) compared with the other user profiles. This group also showed improvements in social functioning (*F*_1,62_=4.68; *P*=.03), negative symptoms (*F*_1,62_=14.61; *P*<.001), and overall psychiatric symptom severity (*F*_1,63_=5.66; *P*=.02) compared with the TAU group. Conversely, the *maintained social* group showed increases in anxiety compared with the TAU group (*F*_1,57_=7.65; *P*=.008). No differences were found between the *low use* group and the TAU group on treatment outcomes.

**Conclusions:**

Continued engagement with both therapy and social components might be key in achieving long-term recovery. Maintained social use and low use outcomes were broadly comparable with TAU, emphasizing the importance of maintaining engagement for improved treatment outcomes. Although the social network may be a key ingredient to increase sustained engagement, as users engaged with this more consistently, it should be leveraged as a tool to engage young people with therapeutic content to bring about social and clinical benefits.

## Introduction

### Background

Evidence indicates the efficacy of specialist early intervention (SEI) services for first-episode psychosis (FEP) in achieving symptomatic remission during the first 2 years of treatment [1,2]. Despite this, the critical period for relapse extends to 5 years from the onset of psychosis, with 55% to 70% of individuals relapsing after 2 years [3,4]. Research has indicated that some treatment effects may not be sustained at 5 years, after 2 years of SEI has ceased [5,6].

Recently, 2 clinical trials addressed these limitations by evaluating the effects of extending SEI up to 5 years. Malla et al [7] found that clinical gains, in terms of remission of positive and negative psychotic symptoms, may be sustained if lower-intensity SEI is offered for an additional 3 years (on top of the 2 years already provided). However, findings from the trial by Albert et al [8] failed to demonstrate any additional benefits from extending SEI by 3 years, but this may be attributable to the high level of treatment provided to control participants in that study.

Although SEI has reported success in improving symptoms during the first 2 years of treatment, many young people with FEP continue to experience poor social and functional outcomes [9,10]. Although social and functional recovery is regarded by young people as the most important aspect of recovery [11], few FEP interventions have made this a primary target [12]. Fowler et al [10] addressed this by evaluating the effectiveness of social therapy in combination with early intervention services, with findings showing increases in structured activity, indicative of improved social functioning after 9 months. Furthermore, a randomized controlled trial (RCT) by Chang et al [13] found improvements in functional outcomes when SEI was extended by 1 year, but this was not sustained at 1- and 2-year follow-ups. Therefore, further research is needed to establish the effectiveness of longer-term interventions focusing on social and functional outcomes.

Digital interventions for FEP provide a unique opportunity to overcome the current limitations of treatment by providing continuous, engaging, and sustainable support to maintain long-term treatment effects [14]. It has been proposed that digital technologies can enhance care in FEP specifically by increasing access, enhancing current treatment, offering better predictive models, and accounting for clinical heterogeneity [15]. Some studies on the effectiveness of digital interventions for treating FEP and those with more established or sustained psychotic disorders have reported improvements in treatment outcomes such as social functioning [16], positive psychotic symptoms [17], negative psychotic symptoms [18], general psychopathology [18,19], overall psychiatric symptom severity [18], vocational outcomes [20], hallucination severity [19,21], hospital admissions [20,22], subjective well-being [16], social support [17], social connectedness [21], medication adherence [21], depression [1], and stress [14,17].

Although digital interventions have been associated with improved outcomes, they have also been associated with high attrition rates [23], and most do not typically extend beyond a 3-month period to focus on long-term recovery [24-26]. To address these limitations, Alvarez-Jimenez et al [1,27] pioneered a model of multicomponent digital interventions entitled *moderated online social therapy* (MOST). The MOST model integrates the following: (1) interactive psychosocial interventions, (2) social networking, (3) expert clinical moderation, and (4) peer support. To address attrition rates, MOST also aims to enhance long-term engagement by offering a shared, secure, and private social network for young people with similar mental health experiences.

The social networking and therapeutic elements of the MOST model were first applied in Horyzons, a world-first digital intervention aimed at maintaining long-term treatment effects and engagement and to improve social functioning in young people recovering from FEP after receiving 2 years of SEI treatment [1,12]. Strengths and mindfulness-based approaches to therapy were adopted, with the aim of increasing self-efficacy and positive emotions, which have been linked to improved social functioning in psychosis [28,29]. The principles of self-determination theory (SDT) were also used with the aim of improving social functioning through increased intrinsic motivation [30].

A 4-week pilot study investigating the acceptability, safety, and clinical benefits of Horyzons indicated that the intervention was feasible, safe, and engaging and may enhance social connectedness in young people recovering from FEP [1]. An 18-month RCT of Horyzons has recently been completed, which found that Horyzons was effective in improving vocational outcomes and reducing presentations to hospital emergency services and hospital admissions compared with a control group receiving treatment as usual (TAU) [20]. Conversely, there were no differences between groups in social functioning over time. However, as there is limited evidence regarding the effectiveness of multicomponent digital interventions based on system use, it is difficult to determine the core therapeutic components of Horyzons, what outcomes they are associated with, and whether a specific pattern of use leads to improved social functioning in this population.

In line with SDT, multicomponent digital interventions based on the MOST model offer young people a high degree of choice over how and when they engage with the system, which increases flexibility in use. This increased flexibility increases the possibility of variation in use patterns or user trajectories. Distinct user profiles may exist in such multicomponent digital interventions, with users who may differ in use and engagement levels over time. The introduction of additional components, such as a therapeutic social network, is needed to address high attrition rates, increase engagement and tailor interventions to cater to the clinical needs and preferences of young people. However, new methods are needed to understand the complexities associated with determining which aspects and patterns of use lead to improved outcomes. Statistical modeling techniques such as growth mixture modeling can be used to identify different groups of users with similar trajectories over time. These techniques have previously been used for detecting similar symptom trajectories in mental health interventions [31]. *K*-means clustering techniques have also been used to identify and characterize participants based on unidimensional and multidimensional trajectories [32-34].

### Objectives

Horyzons provides a unique opportunity to examine the relationship between multidimensional patterns of use and treatment outcomes by categorizing the use of multiple intervention components, such as therapeutic and social networking components. By gaining a better understanding of system use and user trajectories, and how they relate to treatment outcomes, multicomponent digital interventions could be further optimized to improve long-term recovery. Therefore, this study aims to examine the association between user profiles and treatment outcomes on Horyzons by (1) identifying user profiles based on 2D patterns of system use on both therapeutic and social components of the intervention, (2) characterizing the user profiles based on baseline demographic and clinical characteristics, and (3) examining the predictive value of the user profiles for treatment outcomes.

## Methods

### Study Design

Horyzons was a single-blind 18-month RCT, where participants with remitted FEP were randomly allocated to either TAU following 2 years of specialized care or TAU along with access to a moderated web-based social therapy intervention (Horyzons) [12]. Horyzons was based on the MOST model, which integrates (1) web-based therapy (*Pathways and Steps*), (2) peer-to-peer web-based social networking (*the Café*), (3) peer moderation, and (4) expert support by mental health clinicians and vocational workers. This RCT was registered on the Australian New Zealand Clinical Trials Registry (ACTRN12614000009617).

### Ethics Approval

Ethical approval for the Horyzons RCT was granted by the Melbourne Health Research Ethics Committee (2013.146).

### Participants

Participants comprised 86 young people allocated to the Horyzons intervention and 84 young people allocated to TAU, recruited from the Early Psychosis Prevention and Intervention Centre (EPPIC) at Orygen Youth Health, Melbourne, between October 2013 and January 2017. EPPIC is a specialist FEP program that provides 1 ½ to 2 years of specialized care to young people aged 15 to 24 years with FEP [35,36].

A total of 4 intervention participants who did not use the Horyzons platform independently and subsequently had no valid system use data were excluded from analyses. The remaining 82 intervention participants were aged between 16 and 27 years at randomization (mean 21, SD 2.88 years), and the 84 TAU participants were also aged between 16 and 27 years at randomization *(*mean 21, SD 2.83 years). Participants met clinical diagnosis for a first-episode psychotic disorder or mood disorder with psychotic features according to the Diagnostic and Statistical Manual of Mental Disorders 4th Edition (DSM-IV) [37], had not been treated with antipsychotic medication for >6 months before attending EPPIC, and showed remission of positive symptoms of psychosis for ≥4 weeks at the time of enrollment in the Horyzons study, as measured using the Positive and Negative Syndrome Scale [38].

### Measures

#### Demographic and Clinical Characteristics

Data from baseline and the 6-month follow-up were used for this study’s analysis. Demographic information collected at baseline included sex, age, and vocational status. Baseline clinical characteristics included psychotic symptoms, levels of social functioning, depression, and anxiety, and are described in the *Social Functioning*, *Psychotic Symptoms*, and *Depression and Anxiety* sections below.

#### Social Functioning

Social functioning was measured using the Personal and Social Performance Scale (PSP) [39]. Ratings are based on functioning in the following four domains: (1) socially useful activities, (2) personal and social relationships, (3) self-care, and (4) disturbing and aggressive behaviors. The following four subscales of the First Episode Social Functioning Scale (FESFS) were also included to capture the full construct of social functioning: (1) living skills, (2) friends and activities, (3) intimacy, and (4) interacting with people [40]. These subscales were chosen based on their strong psychometric properties, independence from psychotic symptoms, and sensitivity to treatment effects [12]. The FESFS was designed specifically for young people with FEP.

#### Psychotic Symptoms

The Positive and Negative Syndrome Scale was used to assess psychotic symptoms, which included three subscales measuring (1) positive symptoms, (2) negative symptoms, and (3) general psychopathology [38]. The total score comprised all items from the three subscales, which indicated overall psychiatric symptom severity.

#### Depression and Anxiety

Depression was assessed using the Calgary Depression Scale for Schizophrenia [41]. Anxiety was assessed using the Depression Anxiety and Stress Scale [42].

#### System Use Metrics

System use metrics were extracted from the Horyzons web-based platform for each user for each day of their trial involvement. Textbox 1 shows an overview of metrics representing aspects of use of the intervention’s therapeutic and social components.

**System use variables extracted from the Horyzons platform.**

**Therapy-related variables**

- Number of steps started (*steps* refer to the intervention modules)
- Number of actions done (*actions* refer to the activities that comprise a step)
- Visited suggested content (*suggested content* refers to the therapeutic content recommended by clinical moderators)
- Visited therapy (*visiting therapy* refers to visiting the homepage of the therapy component of the intervention)

**Social networking–related variables**

- Number of newsfeed posts (*newsfeed* refers to the social network)
- Number of newsfeed comments
- Number of Talk it Out posts (*Talk it Out* refers to a problem-solving forum run by peer moderators)
- Number of likes made
- Number of reactions made (*reactions* refer to short support messages in response to a post, eg, “thinking of you”)
- Visited messages (*messages* refer to a private message section where moderators could contact participants directly)
- Visited notifications
- Visited newsfeed
- Visited Talk it Out

Therapeutic *Pathways* were divided into themes including understanding psychosis, identifying early warning signs to prevent relapse, identifying and exercising personal strengths, promoting social connections and positive emotions, and managing stress, anxiety, and depression. To increase usability, *Pathways* were further divided into short interactive *Steps*, for example, illustrating how to respond empathically to others (to foster positive connections). See Multimedia Appendix 1 for an example of a *Step* on Horyzons. Each *Step* was accompanied by *Actions* or *Do its*, aiming to translate learning into behavior change, for example, suggestions on how to exercise empathy in specific contexts. Expert clinical moderators could also recommend *Pathways*, *Steps*, *Actions*, and *Talk it Outs* they felt would be relevant to different users via a private message, which would appear as a notification on the user’s dashboard. Furthermore, users could visit the therapeutic component of Horyzons without completing any therapeutic content, for example, viewing what *Pathway* and *Step* was currently allocated to them.

The social network or *the Café* was led and moderated by *peer-workers*, who were trained young people who had a lived experience of mental illness. Participants were encouraged to communicate with one another to foster social support. Participants could post comments on the *Newsfeed* or like, respond, or react to comments that were already posted. Predeveloped *reactions* were designed to facilitate social support, for example, “I get you” and “thinking of you.” Furthermore, participants could use the *Talk It Out* function to nominate relevant issues to discuss in a moderated forum, informed by an evidence-based problem-solving framework [43]. Participants received notifications when other users communicated on the social network. Participants also received private messages when a moderator contacted them directly via the platform. See Multimedia Appendix 2 for an example of a newsfeed post with *likes* and *reactions* on the Horyzons social network.

#### Daily Activity Categories

**Figure 1:**
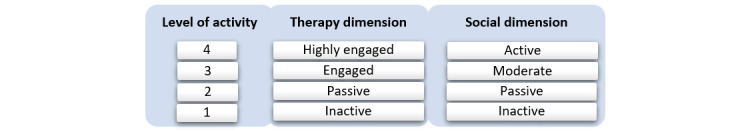
Hierarchical 2D daily activity categories.

For this study, daily activity for both the therapeutic and social networking components was categorized. Daily activity was defined as the hierarchical level of system use per user per day based on the system use variables outlined in Textbox 1. A user was *inactive* when they did not display any activity on either the therapeutic or social component of Horyzons. Use was deemed *passive* when a user visited pages but did not actively engage with any content on either intervention component. A user was *engaged with therapy* when they started 1 step or completed 1 action. A user was *highly engaged with therapy* when they started >1 step, completed >1 action, or started at least one step and completed at least one action. Social use was deemed *moderate* when a user did not actively contribute to the social network but liked or reacted to at least one item. A user *was active* on the social network when they actively contributed via a post or comment. Figure 1 shows the hierarchical categorization of daily activity into 2 dimensions.

### Statistical Analyses

#### Identifying User Profiles

*K*-means clustering for joint trajectories was implemented using the *R* package *kml3d* (R Studio) to identify data-driven user profiles using Euclidean distance [44-46]. This is an unsupervised nonparametric technique that simultaneously partitions user trajectories from both the social and therapy dimensions into distinct cluster groups. This technique uses a hill-climbing expectation-maximization algorithm, alternating through various initialization methods until convergence is reached [45,47].

To run this analysis, participant trajectories were required to be of the same length. As such, we focused on the maximum number of days that all participants used Horyzons, which was 154 days. Each user’s first day on Horyzons consisted of an induction to the platform, so it could not be viewed as an independent system use. Therefore, daily activity (as per the hierarchical categories on both the social and therapeutic components of the intervention) from days 2 to 155 was used.

In terms of adherence, it was expected that participants would use Horyzons fortnightly to benefit from the intervention. On this basis, daily activity was transformed into 22 meaningful weekly scores, and these 22 weekly use scores were used as input features for the *K*-means clustering. Weekly scores comprised the maximum level of use per week, for example, if a user used Horyzons twice during week 1, and this consisted of passive use of the therapy dimension for 1 day (level of activity=2) and engaging with therapy on the second day (level of activity=3); they would obtain a score of 3 for week 1 on the therapy dimension (ie, the highest level of activity in that week).

No a priori hypothesis existed to substantiate the optimal number of clusters for analysis. Therefore, 2- to 4-cluster solutions were examined to account for complex patterns of system use found outside of a dichotomous high versus low use range. Furthermore, the sample was relatively small (N=82), suggesting that cluster solutions exceeding 4 would comprise too few participants per cluster. Cluster solutions with <15 participants in any cluster were excluded. *K*-means was rerun 100 times, each with different initial configurations, to ensure a global maximum was reached. A number of nonparametric fit indexes were used to compare cluster solutions, including the criteria developed by Calinski and Harabasz [48], Ray and Turi [49], and Davies and Bouldin [50]. A higher Calinski and Harabasz score indicates better fit, whereas lower Davies and Bouldin and Ray and Turi scores indicate better fit. In addition, cluster solutions were internally validated by calculating a Rand index, with scores closer to 1 indicating a higher likelihood of being assigned to the same cluster upon running 100 resamples [51]. Theoretical justifications and interpretability were also considered to select the optimal cluster solution.

#### Characterizing User Profiles

Differences between user profiles on demographic and baseline clinical characteristics were investigated using the 1-way analyses of variance and chi-square (*χ*^2^) tests for categorical variables.

#### Examining the Predictive Value of User Profiles for Treatment Outcomes

General linear mixed models were used to assess the associations between user profiles and treatment outcomes using the R package *lme4* [46,52]. Cluster group (user profile), time (baseline, 6-month follow-up), and group-by-time interaction were added as predictors. The predictive value of user profiles was assessed for social functioning, psychotic symptoms, depression, and anxiety. Sex, age, and days of untreated psychosis were added to the models as a priori determined covariates, as shorter days of untreated psychosis has been associated with improved outcomes and remission in FEP [53-58], male sex has been associated with poorer social and functioning outcomes in FEP [53], and age and sex may influence the use of the system. The models also controlled for baseline differences in the outcomes of interest. The effects of interest included (1) the main effect of group, (2) the main effect of time, and (3) the interaction between group and time. User IDs were added to the models as a random intercept effect, as they resolve the nonindependence associated with having multiple responses per user. As a secondary analysis, general linear mixed models were used to assess the associations between each individual user profile and TAU.

## Results

### Clusters Based on Joint Trajectories of System Use

The fit indexes for 2-, 3-, and 4-cluster solutions are reported in Multimedia Appendix 3. The 2-cluster solution was optimal based on all criteria, except for the Bayesian Information Criterion, where the 3-cluster solution showed the best solution. Cluster A (high-decreasing use) and B (low-decreasing use) trajectories remained consistent in the 2-, 3-, and 4-cluster solutions. The 2-cluster solution represented high-decreasing versus low-decreasing use, whereas the 3- and 4-cluster solutions represented more complex intermittent use, which existed based on visual inspections of the data (individual plots available upon request). Cluster C represented more intermittent and consistent use and added valuable information beyond high versus low use in terms of alternative user trajectories. On the basis of these observations, the 3-cluster solution was selected as it was superior to the 4-cluster solution on all fit indexes, and the fit indexes were still relatively high compared with the 2-cluster solution. The 3-cluster solution also showed good internal validity based on the Rand index.

**Figure 2:**
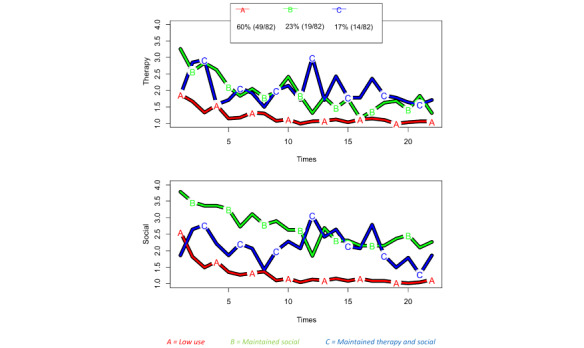
User profile trajectories identified based on weekly hierarchical daily activity scores.

The trajectories of the user profiles based on the 3-cluster solution are shown in Figure 2. User profile A showed a rapid decrease in use on both the social and therapy dimensions after baseline and remained inactive for the following months; hence, this user profile was termed *low use.* User profile B showed initial high use on both dimensions, which decreased over time, with users remaining more active on the social dimension than on the therapy dimension (where use was mainly passive or inactive); therefore, user profile B was called *maintained social.* User profile C showed more variable but sustained use over time, remaining active on both dimensions, except during the final few weeks. User profile C also remained more engaged with the system’s therapy components than the other 2 user profiles and hence was called *maintained therapy and social*. The *low use* profile comprised 60% (49/82) of the users, the *maintained social* profile comprised 23% (19/82) of the users, and the *maintained therapy and social* profile comprised 17% (14/82) of the users.

### Characteristics of User Profiles

A 1-way between-groups analysis of variance indicated a statistically significant difference between user profiles on negative psychotic symptoms at baseline (*F*_2, 79_=6.375; *P*=.003). Post hoc comparisons using the Tukey honest significant difference test indicated that the *maintained therapy and social* profile (mean 14.36, SD 4.99) had significantly higher symptoms than the *maintained social* profile (mean 10.48, SD 3.14) and the *low use* profile (mean 11.05, SD 3.55). No significant differences were observed between user profiles on any other clinical characteristics or on any demographic variables at baseline. A full overview of the results can be found in Multimedia Appendix 4.

### Associations Between User Profiles and Treatment Outcomes

**Figure 3:**
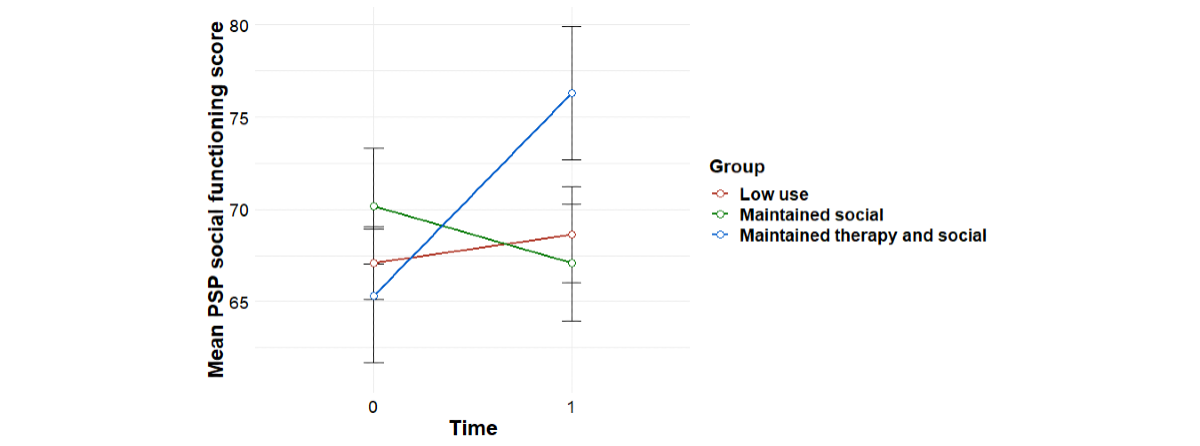
Mean trends in PSP social functioning scores for user profiles (95% CIs). PSP: Personal and Social Performance Scale.

**Figure 4:**
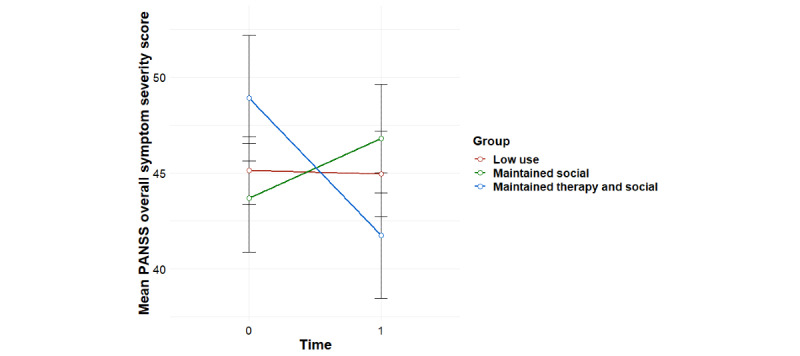
Mean trends in PANSS overall psychiatric symptom severity scores for user profiles (95% CIs). PANSS: Positive and Negative Syndrome Scale.

**Figure 5:**
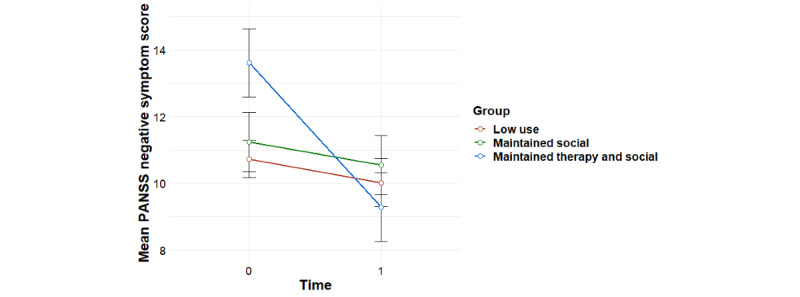
Mean trends in PANSS negative symptom scores for user profiles (95% CIs). PANSS: Positive and Negative Syndrome Scale.

Significant group-by-time interaction effects were found for the primary outcome of social functioning as measured using the PSP, overall psychiatric symptom severity, and negative psychotic symptoms, with baseline effects accounted for (Table 1). Post hoc tests revealed that for social functioning, this interaction effect was accounted for by significant improvements for the *maintained therapy and social* profile from 0 to 6 months (*F*_1,11_=8.81; *P*=.01), compared with the *maintained social* profile (*F*_1,15_=1.35; *P*=.26) and the *low use* profile (*F*_1,28_=0.17; *P*=.68; Figure 3). For overall psychiatric symptom severity, post hoc tests revealed that this interaction effect was accounted for by a significant decrease in symptoms for the *maintained therapy and social* profile (*F*_1,11_=5.99; *P*=.03), compared with the *maintained social* profile (*F*_1,15_=1.71; *P*=.21) and the *low use* profile (*F*_1,27_=0.004; *P*=.95; Figure 4). In terms of negative symptoms, post hoc tests revealed that this interaction effect was accounted for by a significant reduction in symptoms for the *maintained therapy and social* profile (*F*_1,11_=10.94; *P*=.007), compared with the *maintained social* profile (*F*_1,15_=0.66; *P*=.43) and the *low use* profile (*F*_1,27_=0.98; *P*=.33; Figure 5).

**Table 1:** Changes on outcomes from baseline to 6 months for user profiles.

		User profiles				Group × time interaction, *F* test (*df*)		*P* value
		Low use (n=49), mean (SD)	Maintained social (n=19), mean (SD)	Maintained therapy and social (n=14), mean (SD)				
**PSP^a^**								
	Baseline	67.09 (1.97)	70.17 (3.15)	65.31 (3.64)	N/A^b^		N/A	
	6 months	68.64 (2.62)	67.11 (3.15)	76.31 (3.64)	3.58 (2, 51)		.04	
**FESFS^c^ independent living skills**								
	Baseline	13.66 (0.33)	14.07 (0.52)	13.11 (0.60)	N/A		N/A	
	6 months	13.59 (0.41)	14.04 (0.54)	13.03 (0.62)	0.003 (2, 45)		.99	
**FESFS interacting with people**								
	Baseline	12.96 (0.35)	12.52 (0.56)	11.90 (0.64)	N/A		N/A	
	6 months	13.26 (0.44)	12.09 (0.58)	12.27 (0.66)	0.87 (2, 45)		.42	
**FESFS friends and activities**								
	Baseline	18.96 (0.47)	17.50 (0.75)	17.61 (0.86)	N/A		N/A	
	6 months	19.53 (0.62)	17.70 (0.78)	18.06 (0.89)	0.08 (2, 46)		.92	
**FESFS intimacy**								
	Baseline	15.37 (0.45)	15.22 (0.75)	.52 (0.85)	N/A		N/A	
	6 months	15.24 (0.58)	14.77 (0.75)	13.52 (0.90)	0.43 (2, 40)		.65	
**PANSS^d^ total**								
	Baseline	45.15 (1.77)	43.69 (2.84)	48.91 (3.27)	N/A		N/A	
	6 months	44.96 (2.22)	46.80 (2.84)	41.73 (3.27)	3.23 (2, 50)		.048	
**PANSS positive**								
	Baseline	10.49 (0.54)	10.38 (0.86)	10.31 (1.00)	N/A		N/A	
	6 months	10.29 (0.70)	11.31 (0.86)	9.89 (1.00)	0.60 (2, 52)		.55	
**PANSS negative**								
	Baseline	10.73 (0.55)	11.24 (0.89)	13.62 (1.03)	N/A		N/A	
	6 months	10.02 (0.72)	10.55 (0.89)	9.28 (1.03)	4.45 (2, 51)		.02	
**PANSS general psychopathology**								
	Baseline	23.93 (1.06)	22.07 (1.70)	24.99 (1.96)	N/A		N/A	
	6 months	24.65 (1.33)	24.92 (1.70)	22.56 (1.96)	2.34 (2, 50)		.11	
**CDSS^e^**								
	Baseline	3.91 (0.67)	3.31 (1.08)	2.78 (1.25)	N/A		N/A	
	6 months	4.41 (0.84)	3.84 (1.08)	2.20 (1.25)	0.33 (2, 50)		.72	
**DASS^f^ anxiety**								
	Baseline	12.14 (1.35)	6.79 (2.02)	10.15 (2.45)	N/A		N/A	
	6 months	12.02 (1.77)	10.58 (2.18)	9.42 (2.53)	1.35 (2, 42)		.27	

^a^PSP: Personal and Social Performance Scale.

^b^N/A: not applicable.

^c^FESFS: First Episode Social Functioning Scale.

^d^PANSS: Positive and Negative Syndrome Scale.

^e^CDSS: Calgary Depression Scale for Schizophrenia.

^f^DASS: Depression, Anxiety, and Stress Scale.

No significant group (user profile) by time associations were found for aspects of social functioning as measured by the FESFS, positive psychotic symptoms, general psychopathology, depression, or anxiety (Table 1). Furthermore, no main effects were found for differences between the profiles at each time point, and no main effects were found for changes over time for each profile on the outcomes.

### Associations Between Individual User Profiles and TAU With Treatment Outcomes

**Figure 6:**
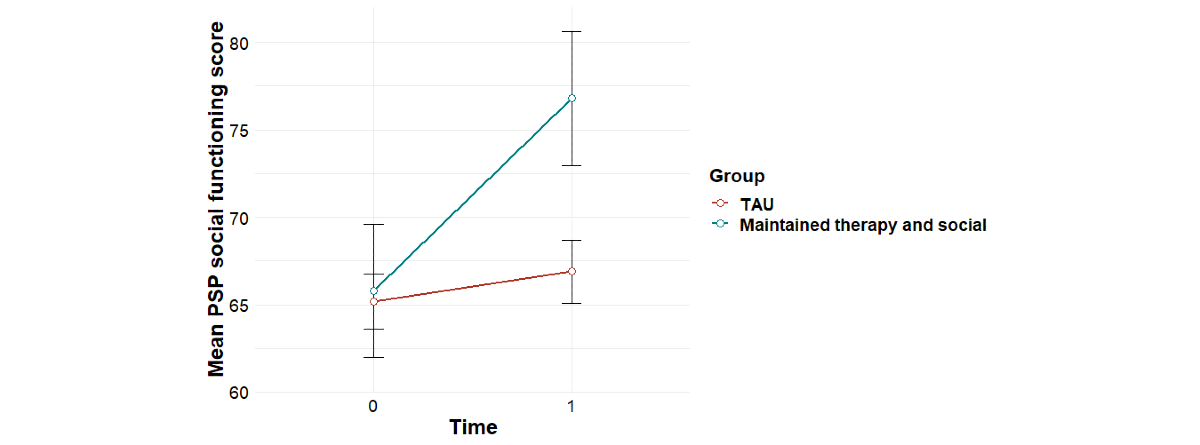
Mean trends in PSP social functioning scores for the maintained therapy and social and TAU groups (95% CIs). PSP: Personal and Social Performance Scale; TAU: treatment as usual.

**Figure 7:**
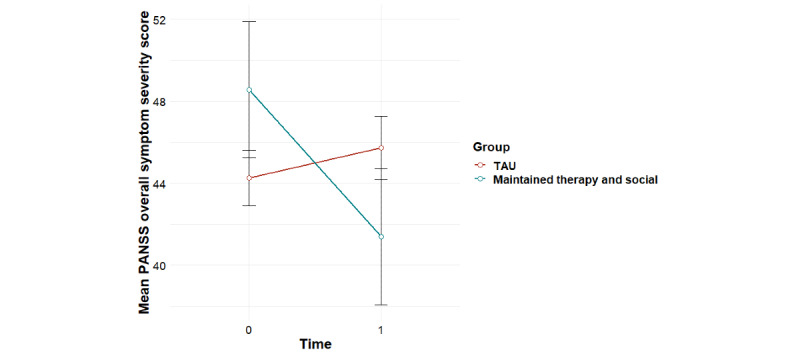
Mean trends in PANSS overall psychiatric symptom severity scores for the maintained therapy and social and TAU groups (95% CIs). PANSS: Positive and Negative Syndrome Scale; TAU: treatment as usual.

**Figure 8:**
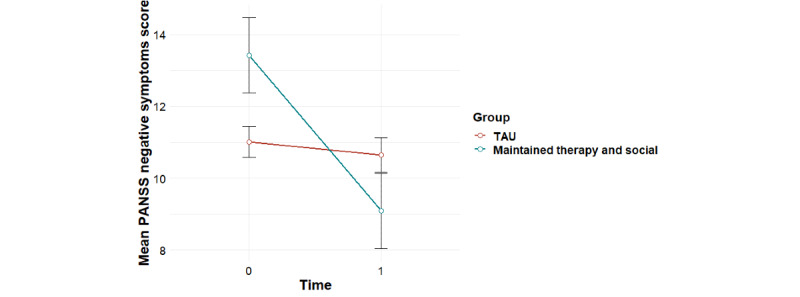
Mean trends in PANSS negative symptom scores for the maintained therapy and social and TAU groups (95% CIs). PANSS: Positive and Negative Syndrome Scale; TAU: treatment as usual.

Significant group-by-time interaction effects were found for social functioning as measured using the PSP, overall psychiatric symptom severity, and negative psychotic symptoms for the *maintained therapy and social* group versus the *TAU* group (Table 2). For social functioning, post hoc tests revealed that this interaction effect was accounted for by improvements for the *maintained therapy and social* group (*F*_1,11_=8.81; *P*=.01) compared with the *TAU* group (*F*_1,56_=0.87; *P*=.35), and a significant difference between groups at 6 month follow-up, with the *maintained therapy and social* group having higher social functioning scores than the *TAU* group (*F*_1,57_=5.82; *P*=.02; Figure 6). In terms of overall psychiatric symptom severity, post hoc tests revealed that this interaction effect was accounted for by decreases in symptoms for the *maintained therapy and social* group (*F*_1,11_=5.99; *P*=.03), compared with the *TAU* group (*F*_1,57_=0.78; *P*=.38; Figure 7). In terms of negative symptoms, post hoc tests revealed that this interaction effect was accounted for by decreases in symptoms for the *maintained therapy and social* group (*F*_1,11_=10.94; *P*=.006) compared with the *TAU* group (*F*_1,54_=0.71; *P*=.40), and a significant difference between groups at baseline, with the *maintained therapy and social* group having higher symptoms than the *TAU* group (*F*_1,76_=4.35; *P*=.04; Figure 8).

**Figure 9:**
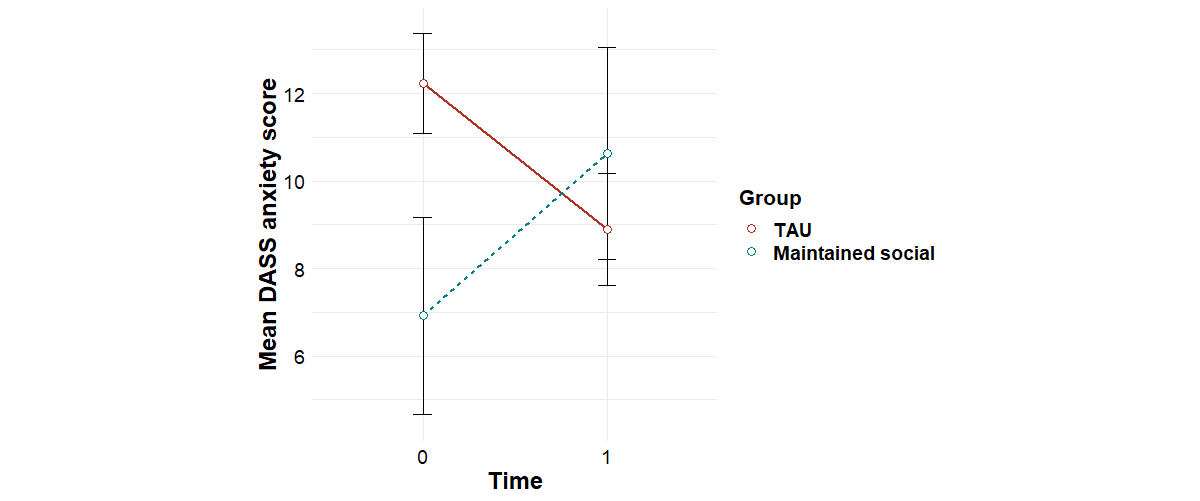
Mean trends in DASS anxiety scores for the maintained social and TAU groups (95% CIs). DASS: Depression Anxiety and Stress Scale; TAU: treatment as usual.

No significant group-by-time associations were found for treatment outcomes for the *low use* group versus the *TAU* group (Multimedia Appendix 5). Similarly, with the exception of anxiety, no significant group-by-time associations were found for treatment outcomes for the *maintained social* group versus *the TAU* group (Multimedia Appendix 6). Post hoc tests revealed that the interaction effect found for anxiety was accounted for by a significant decrease in symptoms for the *TAU* group (*F*_1,47_=6.50; *P*=.01), a significant increase in symptoms for the *maintained social* group (*F*_1,12_=6.11; *P*=.03), and a significant difference between groups at baseline, with the *maintained social* group having lower anxiety scores than the *TAU* group (*F*_1,72_=4.07; *P*=.047; Figure 9).

**Table 2:** Changes on outcomes from baseline to 6 months for the maintained therapy and social and TAU^a^ groups.

		TAU (n=84), mean (SD)	Maintained therapy and social (n=19), mean (SD)	Group × time interaction, *F* test (*df*)	*P* value
**PSP^b^**					
	Baseline	65.19 (1.57)	65.79 (3.82)	N/A^c^	N/A
	6 months	66.89 (1.80)	76.79 (3.82)	4.68 (1, 62)	.03
**FESFS^d^ independent living skills**					
	Baseline	13.71 (0.21)	13.12 (0.50)	N/A	N/A
	6 months	13.71 (0.24)	13.04 (0.51)	0.02 (1, 60)	.88
**FESFS interacting with people**					
	Baseline	12.78 (0.24)	11.94 (0.58)	N/A	N/A
	6 months	12.62 (0.27)	12.31 (0.59)	0.99 (1, 57)	.32
**FESFS friends and activities**					
	Baseline	18.47 (0.43)	17.74 (1.00)	N/A	N/A
	6 months	18.25 (0.47)	18.20 (1.02)	0.60 (1, 55)	.44
**FESFS intimacy**					
	Baseline	14.82 (0.39)	14.56 (0.92)	N/A	N/A
	6 months	14.77 (0.42)	13.47 (0.96)	1.63 (1, 52)	.20
**PANSS^e^ total**					
	Baseline	44.26 (1.37)	48.57 (3.32)	N/A	N/A
	6 months	45.72 (1.54)	41.40 (3.32)	5.66 (1, 63)	.02
**PANSS positive**					
	Baseline	9.47 (0.43)	10.24 (1.05)	N/A	N/A
	6 months	9.72 (0.50)	9.82 (1.05)	0.24 (1, 64)	.62
**PANSS negative**					
	Baseline	11.00 (0.43)	13.42 (1.05)	N/A	N/A
	6 months	10.64 (0.48)	9.09 (1.05)	14.61 (1, 62)	<.001
**PANSS general psychopathology**					
	Baseline	23.78 (0.83)	24.92 (2.01)	N/A	N/A
	6 months	25.39 (0.95)	22.50 (2.01)	2.76 (1, 63)	.10
**CDSS^f^**					
	Baseline	2.72 (0.41)	2.86 (0.99)	N/A	N/A
	6 months	3.46 (0.46)	2.29 (0.99)	1.56 (1, 62)	.22
**DASS^g^ anxiety**					
	Baseline	12.35 (1.16)	8.85 (2.80)	N/A	N/A
	6 months	9.00 (1.32)	8.21 (2.89)	0.76 (1, 52)	.39

^a^TAU: treatment as usual.

^b^PSP: Personal and Social Performance Scale.

^c^N/A: not applicable.

^d^FESFS: First Episode Social Functioning Scale.

^e^PANSS: Positive and Negative Syndrome Scale.

^f^CDSS: Calgary Depression Scale for Schizophrenia.

^g^DASS: Depression, Anxiety, and Stress Scale.

## Discussion

### Principal Findings

This is the first study that characterized participants’ patterns of use of a multicomponent digital intervention (Horyzons), which included interactive therapy content and social networking, to predict treatment outcomes for young people recovering from FEP. Using a clustering procedure for joint trajectories, we identified three distinct user profiles: (1) low use, (2) maintained use of social components (*maintained social*), and (3) maintained use of both therapy and social components (*maintained therapy and social*). The *maintained therapy and social* profile had higher negative symptoms at baseline compared with the *maintained social* and *low use* profiles. The *maintained therapy and social* profile showed statistically significant improvements in social functioning and decreases in negative symptoms and overall psychiatric symptom severity compared with both the low use and maintained social profiles and the TAU group.

We used *K*-means clustering for joint trajectories to identify user profiles beyond that of a high versus low use dichotomy. Our approach accounted for the level of activity over time across the intervention components, going beyond categorizing the number of log-ins, which is limited in terms of meaningful engagement. In doing so, we found that the user profiles of individuals who demonstrated more variable but sustained use of both the therapy and social components over time were significantly associated with improved social functioning and clinical outcomes. In contrast, user profiles consisting of individuals with decreasing use on both social and therapy dimensions (ie, low use) demonstrated clinical outcomes comparable with that of TAU. The use of *K*-means clustering to identify use patterns in digital mental health interventions in the literature is a novel approach. A recent study by Sanatkar et al [59] used it to examine the association between engagement profiles (based on 2-month system use metrics) and depression and anxiety outcomes. They reported overall reductions in depressive and anxiety symptoms, but no differences were observed between the clusters. However, all users were somewhat engaged during this 2-month period, making it difficult to determine the optimal levels of use for improved treatment outcomes and how this was compared with nonuse. These findings differ from other research indicating that dropout rates in mental health apps are very rapid during the first month, with a retention rate of only 3.3% in the general population [60] and 0.5% to 28.6% completion rates or use beyond 6 weeks in interventions targeting depression and anxiety [61]. However, little is known about use patterns beyond a 2-month period, which our study examined.

Our findings indicated that the maintained use of both the therapy and social components was significantly associated with improvements in social functioning (the primary outcome of the Horyzons trial) compared with the other 2 user profiles. This is consistent with a recent pilot study investigating the effectiveness of a strengths- and mindfulness-based web-based social therapy for young people at ultrahigh risk of psychosis, which found increases in social functioning at 2-month follow-up [16]. It is worth noting that the MOST platform design is informed by SDT [62], which emphasizes meeting three key psychological needs to support motivation and behavioral change: (1) autonomy (feeling a sense of choice about one’s behavior), (2) competence (being able to bring about positive changes in desired outcomes), and (3) relatedness (feeling accepted by one’s social milieu). It may be the case that the combined system use (eg, the *maintained therapy and social* profile) aligned with both competence (therapy) and relatedness (social network), providing support for the SDT framework as a potentially mediating means in which to improve social functioning outcomes. Young people could engage in therapy on their own terms, which may also have promoted competence and autonomy. For example, choice in treatment (such as the choice young people had to complete therapy they felt was relevant to their needs on Horyzons) has been tied to the notion of individual autonomy [63]. Furthermore, there is evidence to suggest that moderated therapy, such as that offered on Horyzons, can promote self-competence in young people in particular [64].

Other improved outcomes, in terms of negative symptoms and overall psychiatric symptom severity, were observed for the *maintained therapy and social* group compared with the other 2 user profiles on Horyzons. These findings are consistent with those of the Horyzons RCT, which reported lower levels of negative symptoms compared with TAU from baseline to 12 months (which corresponded with a period of higher use of the Horyzons platform) [20], and lends support to the notion that Horyzons may improve negative symptoms for those young people with a certain level of engagement with the digital platform.

To be able to improve outcomes, our study suggests that sustained engagement with both the therapy and social networking components of Horyzons is required. Although these users comprised only 17% (14/82) of our sample, social functioning and negative symptoms are typically treatment resistant in FEP, which highlights the clinical significance of this finding [65,66]. Overall, 40% (33/82) of the users showed sustained use either on the social network alone (19/82, 23%) or on both the therapy and social networking aspects of the intervention (14/82, 17%), whereas 60% (49/82) of the participants were in the *low use* profile. This is an important observation, as a recent systematic search indicated a 15-day retention rate of 3.9% and a 30-day retention rate of 3.3% for mental health apps [60]; in contrast, 40% (33/82) of the users in our study showed more sustained use over 155 days. Furthermore, it is important to note that the *low use* profile did not mean *nonuse.* This cluster had a mean number of 12 log-ins to Horyzons over 6 months, indicating that these young people did engage with Horyzons but to a lesser extent than the *maintained social* (mean log-ins 142) and *maintained therapy and social* (mean log-ins 75) profiles. This also indicates that log-ins are not a good indicator of intervention effectiveness, as the *maintained social* profile had a higher number of log-ins than the *maintained therapy and social* profile but lower consistent engagement with therapy content. Therefore, rather than designing platforms to maximize log-ins, we need to design platforms to promote sustained engagement with the therapy and social components.

Exploratory analyses comparing each user profile to TAU further supported our main findings by demonstrating statistically significant improvements in social functioning, negative symptoms, and overall psychiatric symptom severity for the *maintained therapy and social* group compared with the TAU group. Conversely, increases in anxiety were observed for those in the *maintained social* group compared with those in the TAU group. An explanation for this may be that worsening of outcomes may lead to increased motivation to engage with social aspects and low motivation or perceived competence to engage with therapeutic content. Use of the social network was mostly moderate, with users mostly liking and reacting to posts (indicating passive use) rather than actively contributing via a post or comment. These findings are consistent with a study that found passive social media use to be associated with increased anxiety among adolescents [67]. Therefore, although the social network may increase engagement, as users engaged with this more consistently on Horyzons, it is important that it is designed to reduce anxiety and is leveraged to increase young people’s motivation to engage with therapeutic content for continued support, to bring about improved social and clinical outcomes. This is especially important given the critical 5-year period for the risk of relapse in FEP [4].

Our study confirmed that use is complex and that, although the *maintained therapy and social* profile showed improvements in outcomes, they also had higher negative symptoms at baseline. This raises the question of whether higher negative symptoms led to higher engagement, higher engagement led to improvements in outcomes, or both. For example, higher baseline symptoms may relate to perceived need to engage with therapeutic content. This is in line with previous research, which found that certain users only engaged with therapy until they completed what was relevant for them. Pung et al [68] offered participants self-help management strategies, similar to what was offered on Horyzons, for example, mindfulness steps and opportunities for social connection. Participants discontinued use after a skill was acquired but still had access to the intervention in case symptoms reemerged. These various reasons for use and disengagement may mask associations and contribute to mixed findings on the effectiveness of digital interventions. Although we controlled for baseline differences and our findings were consistent across social and clinical outcomes, we cannot make causal inferences about the change in outcomes in our study. Future research could address this by using multilevel models with an autoregressive lag [69] and examining the relationship between patterns of engagement and outcomes in real time [70].

### Limitations

It should also be noted that this study had a number of methodological limitations. Although we controlled for baseline differences and key potential confounders, the findings of these analyses need to be interpreted with caution. First, the analyses comparing each user profile with TAU were exploratory and nonrandomized. Second, owing to the small sample size, we could not correct for multiple comparisons. That said, this is the first study to explore patterns of use in a multicomponent digital intervention for FEP and is arguably an informative starting point, as our results have significant clinical implications. Future research could build upon this contribution to the literature by replicating these analyses with larger sample sizes.

### Conclusions

In conclusion, our findings indicate that sustained engagement with both the therapeutic and social networking components of Horyzons was key in improving social functioning, negative symptoms, and overall psychiatric symptom severity in young people with psychosis. This supports the therapeutic value of Horyzons and points to the need to capture complex patterns of use over time to determine key therapeutic targets and optimal use for improved outcomes. Going forward, this can be done in real time, with ongoing optimization of intervention features and management against key outcomes. This is a development in progress as digital interventions based on the MOST model are currently being implemented into clinical services as part of routine care, and novel methodologies including fast iterative A/B testing and artificial intelligence optimization methods will be used to fast-track innovation and research translation [70]. These findings have real-world implications for the development of multicomponent digital interventions, as well as for the treatment of young people with psychosis through digital platforms. Future research will need to determine how to distill the contribution of specific aspects of the intervention and how components may work together to sustain user engagement and improve clinical outcomes. We are currently investigating this by determining which aspects of Horyzons system use lead to subsequent use by means of multiple convergent cross mapping.

## Appendix

Multimedia Appendix 1 Horyzons step: how to flourish.

Multimedia Appendix 2 Horyzons social network.

Multimedia Appendix 3 Fit indexes for each cluster solution.

Multimedia Appendix 4 Baseline demographic and clinical characteristics for user profiles.

Multimedia Appendix 5 Changes in outcomes from baseline to 6-months for the low use and treatment as usual groups.

Multimedia Appendix 6 Changes in outcomes from baseline to 6-months for the maintained social and treatment as usual groups.

## Abbreviations

EPPIC: Early Psychosis Prevention and Intervention Centre
FEP: first-episode psychosis
FESFS: First Episode Social Functioning Scale
MOST: Moderated Online Social Therapy
PSP: Personal and Social Performance Scale
RCT: randomized controlled trial
SDT: self-determination theory
SEI: specialist early intervention
TAU: treatment as usual
