# Supplementary figures and images for: Characterizing Use of a Multicomponent Digital Intervention to Predict Treatment Outcomes in First-Episode Psychosis: Cluster Analysis
Source: JMIR Ment Health. 2022 Apr 7;9(4):e29211. doi: 10.2196/29211 (PMC9030973; doi:10.2196/29211)

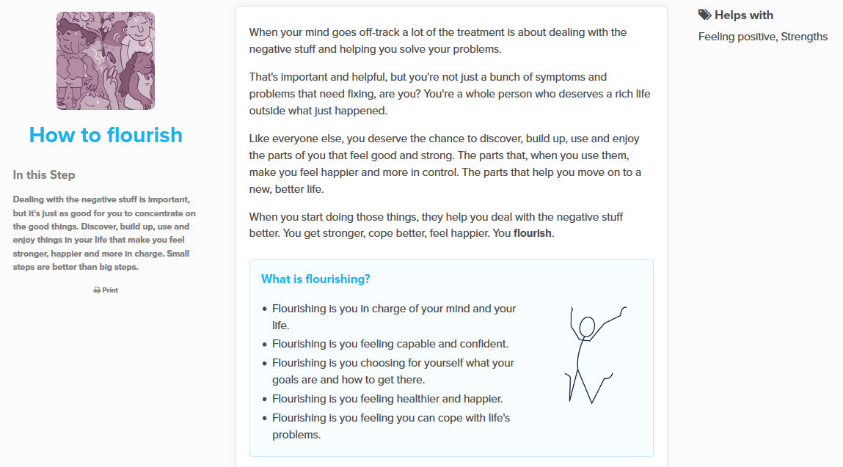

Supplement: Multimedia Appendix 1 [file mental_v9i4e29211_app1.png]

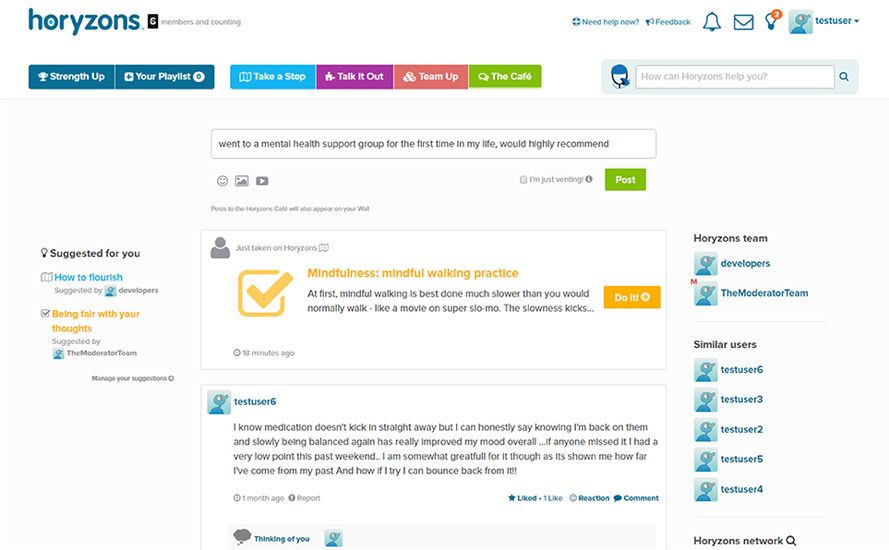

Supplement: Multimedia Appendix 2 [file mental_v9i4e29211_app2.png]
