# Supplementary material for: Characterizing Use of a Multicomponent Digital Intervention to Predict Treatment Outcomes in First-Episode Psychosis: Cluster Analysis
Source: JMIR Ment Health. 2022 Apr 7;9(4):e29211. doi: 10.2196/29211 (PMC9030973; doi:10.2196/29211)
Supplement: Multimedia Appendix 3 [file mental_v9i4e29211_app3.doc]

**Multimedia Appendix 3. Fit indices for each cluster solution.**

|  |  | Quality criteria | | | | |
| --- | --- | --- | --- | --- | --- | --- |
|  |  | CH^a^ | RT^b^ | DB^c^ | BIC | Rand index |
| **2 cluster solution** | A = 63.4%  B = 36.6% | 26.34 | 0.02 | 1.52 | -9398.96 | .923 |
| **3 cluster solution** | A = 59.8%  B = 23.2%  C = 17.1% | 16.98 | 0.03 | 1.74 | -9377.18 | .717 |
| **4 cluster solution** | A = 39%  B = 23.2%  C = 20.7%  D = 17.1% | 13.32 | 0.05 | 1.76 | -9430.43 | .507 |

^a^CH = Calinski and Harabasz criterion; ^b^RT = Ray and Turi criterion; ^c^DB = Davies and Bouldin criterion. Best fit = Higher CH; smaller DB and RT; BIC closer to 0; Rand closer to 1.
