# Supplementary material for: Characterizing Use of a Multicomponent Digital Intervention to Predict Treatment Outcomes in First-Episode Psychosis: Cluster Analysis
Source: JMIR Ment Health. 2022 Apr 7;9(4):e29211. doi: 10.2196/29211 (PMC9030973; doi:10.2196/29211)
Supplement: Multimedia Appendix 4 [file mental_v9i4e29211_app4.doc]

**Multimedia Appendix 4.** Baseline demographic and clinical characteristics for user profiles.

|  | User Profiles | | |  |
| --- | --- | --- | --- | --- |
|  | Low usage (n=49),  mean (SD) | Maintained social (n=19),  mean (SD) | Maintained therapy and social (n=14),  mean (SD) | Statistic |
| **Sex,** n (%)  Male  Female | 25 (51.0)  24 (49.0) | 11 (57.9)  8 (42.1) | 7 (50.0)  7 (50.0) | 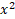 = .300  *P* = .86 |
| Age | 20.61 (3.01) | 21.47 (2.44) | 21.86 (3.03) | *F* = 1.305  *P* = .28 |
| Days of untreated psychosis, median (IQR) | 30 (176) | 67 (454) | 60 (291) | 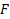= 0.415  *P* = .66 |
| PSP^a^ | 67.14 (14.53) | 69.05 (10.56) | 62.21 (13.13) | *F* = 1.089  *P* = .34 |
| FESFS^b^ Living Skills | 13.58 (2.24) | 14.00 (1.67) | 13.21 (1.76) | *F* = 0.612  *P* = .55 |
| FESFS^b^ Interacting with People | 12.86 (2.24) | 12.26 (2.54) | 11.86 (1.96) | *F* = 1.258  *P* = .29 |
| FESFS^b^ Intimacy | 15.45 (2.58) | 15.29 (2.73) | 14.00 (1.54) | *F* = 1.645  *P* = .20 |
| FESFS^b^ Friends and Activities | 18.74 (3.53) | 17.48 (2.90) | 17.29 (3.15) | *F* = 1.592  *P* = .21 |
| PANSS^c^ Total | 45.22 (11.66) | 44.63 (9.40) | 52.58 (15.38) | *F* = 2.336  *P* = .10 |
| PANSS^c^ Positive | 10.53 (3.08) | 10.47 (2.59) | 11.07 (5.12) | *F* = 0.157  *P* = .86 |
| PANSS^c^ Negative | 10.48 (3.14) | 11.05 (3.55) | 14.36 (4.99) | *F* = 6.375  *P* = .003 |
| PANSS^c^ General Psychopathology | 24.20 (7.50) | 23.11 (6.07) | 27.15 (8.27) | *F* = 1.295  *P* = .280 |
| CDSS^d^ | 4.21 (4.96) | 3.38 (3.46) | 3.06 (3.62) | *F* = 0.483  *P* = .619 |
| DASS Anxiety^e^ | 13.00 (9.73) | 8.62 (9.85) | 10.50 (7.44) | *F* = 1.516  *P* = .226 |

^a^PSP = Personal and Social Performance Scale; ^b^FESFS = First Episode Social Functioning Scale. ^c^PANSS = Positive and Negative Syndrome Scale; ^d^CDSS = Calgary Depression Scale for Schizophrenia; ^e^DASS = Depression, Anxiety and Stress Scale.
