# Supplementary material for: Characterizing Use of a Multicomponent Digital Intervention to Predict Treatment Outcomes in First-Episode Psychosis: Cluster Analysis
Source: JMIR Ment Health. 2022 Apr 7;9(4):e29211. doi: 10.2196/29211 (PMC9030973; doi:10.2196/29211)
Supplement: Multimedia Appendix 5 [file mental_v9i4e29211_app5.doc]

**Multimedia Appendix 5.** Changes in outcomes from baseline to 6-months for the low use and treatment as usual groups.

|  | TAU (n=84),  Mean (SD) | Low usage (n=49),  Mean (SD) | *F* (Group*time interaction) | df | *P* value |
| --- | --- | --- | --- | --- | --- |
| **PSP^a^**  Baseline  6 months | 65.40 (1.65)  67.16 (1.89) | 67.21 (2.14)  68.53 (2.79) | ….  0.02 | ….  1, 87 | ….  .89 |
| **FESFS^b^ Independent Living skills**  Baseline  6 months | 13.71 (0.23)  13.72 (0.26) | 13.61 (0.30)  13.55 (0.39) | ….  0.03 | ….  1, 77 | ….  .86 |
| **FESFS^b^ Interacting with People**  Baseline  6 months | 12.81 (0.24)  12.66 (0.27) | 12.91 (0.31)  13.21 (0.39) | ….  1.17 | ….  1, 74 | ….  .28 |
| **FESFS^b^ Friends and Activities**  Baseline  6 months | 18.52 (0.40)  18.31 (0.45) | 19.00 (0.52)  19.50 (0.65) | ….  1.06 | ….  1, 72 | ….  .30 |
| **FESFS^b^ Intimacy**  Baseline  6 months | 14.84 (0.38)  14.78 (0.41) | 15.35 (0.47)  15.24 (0.56) | ….  0.01 | ….  1, 63 | ….  .91 |
| **PANSS^c^ Total**  Baseline  6 months | 44.12 (1.37)  45.47 (1.55) | 45.19 (1.78)  45.05 (2.27) | ….  0.29 | ….  1, 87 | ….  .59 |
| **PANSS^c^ Positive**  Baseline  6 months | 9.46 (0.40)  9.68 (0.47) | 10.42 (0.52)  10.18 (0.69) | ….  0.23 | ….  1, 96 | ….  .63 |
| **PANSS^c^ Negative**  Baseline  6 months | 10.98 (0.43)  10.62 (0.47) | 10.70 (0.56)  10.02 (0.68) | ….  0.18 | ….  1, 83 | ….  .67 |
| **PANSS^c^ General Psychopathology**  Baseline  6 months | 23.67 (0.84)  25.20 (0.96) | 24.06 (1.09)  24.78 (1.42) | ….  0.20 | …  1, 90 | ….  .66 |
| **CDSS^d^**  Baseline  6 months | 2.72 (0.47)  3.45 (0.54) | 3.88 (0.61)  4.40 (0.78) | ….  0.05 | ….  1, 88 | ….  .82 |
| **DASS Anxiety^e^**  Baseline  6 months | 12.21 (1.18)  8.85 (1.33) | 12.42 (1.55)  12.44 (2.01) | ….  2.16 | …. 1, 69 | ….  .15 |

^a^PSP = Personal and Social Performance Scale; ^b^FESFS = First Episode Social Functioning Scale. ^c^PANSS = Positive and Negative Syndrome Scale; ^d^CDSS = Calgary Depression Scale for Schizophrenia; ^e^DASS = Depression, Anxiety and Stress Scale.
