# Supplementary material for: Characterizing Use of a Multicomponent Digital Intervention to Predict Treatment Outcomes in First-Episode Psychosis: Cluster Analysis
Source: JMIR Ment Health. 2022 Apr 7;9(4):e29211. doi: 10.2196/29211 (PMC9030973; doi:10.2196/29211)
Supplement: Multimedia Appendix 6 [file mental_v9i4e29211_app6.doc]

**Multimedia Appendix 6.** Changes in outcomes from baseline to 6-months for the maintained social and treatment as usual groups.

|  | TAU (n=84),  Mean (SD) | Maintained social (n=19),  Mean (SD) | *F* (Group*time interaction) | df | *P* value |
| --- | --- | --- | --- | --- | --- |
| **PSP^a^**  Baseline  6 months | 65.33 (1.51)  67.06 (1.73) | 70.34 (3.17)  67.28 (3.17) | ….  1.68 | ….  1, 66 | ….  .20 |
| **FESFS^b^ Independent Living skills**  Baseline  6 months | 13.70 (0.21)  13.70 (0.23) | 14.10 (0.43)  14.06 (0.45) | ….  0.01 | ….  1, 63 | ….  .92 |
| **FESFS^b^ Interacting with People**  Baseline  6 months | 12.79 (0.25)  12.63 (0.28) | 12.47 (0.52)  12.04 (0.54) | ….  0.30 | ….  1, 61 | ….  .59 |
| **FESFS^b^ Friends and Activities**  Baseline  6 months | 18.49 (0.42)  18.29 (0.47) | 17.57 (0.85)  17.80 (0.91) | ….  0.26 | ….  1, 59 | ….  .61 |
| **FESFS^b^ Intimacy**  Baseline  6 months | 14.84 (0.40)  14.78 (0.43) | 15.20 (0.82)  14.88 (0.82) | ….  0.18 | ….  1, 54 | ….  .67 |
| **PANSS^c^ Total**  Baseline  6 months | 44.12 (1.34)  45.51 (1.51) | 43.82 (2.81)  46.93 (2.81) | ….  0.29 | ….  1, 67 | ….  .59 |
| **PANSS^c^ Positive**  Baseline  6 months | 9.43 (0.40)  9.65 (0.47) | 10.34 (0.84)  11.28 (0.84) | ….  0.40 | ….  1, 69 | ….  .53 |
| **PANSS^c^ Negative**  Baseline  6 months | 10.97 (0.44)  10.63 (0.48) | 11.34 (0.92)  10.65 (0.92) | ….  0.16 | ….  1, 66 | ….  .69 |
| **PANSS^c^ General Psychopathology**  Baseline  6 months | 23.72 (0.83)  25.28 (0.95) | 22.14 (1.74)  24.99 (1.74) | ….  0.35 | …  1, 68 | ….  .55 |
| **CDSS^d^**  Baseline  6 months | 2.72 (0.42)  3.44 (0.47) | 3.29 (0.88)  3.81 (0.88) | ….  0.05 | ….  1, 66 | ….  .82 |
| **DASS Anxiety^e^**  Baseline  6 months | 12.23 (1.13)  8.89 (1.28) | 6.93 (2.25)  10.64 (2.42) | ….  7.65 | …. 1, 57 | ….  .008 |

^a^PSP = Personal and Social Performance Scale; ^b^FESFS = First Episode Social Functioning Scale. ^c^PANSS = Positive and Negative Syndrome Scale; ^d^CDSS = Calgary Depression Scale for Schizophrenia; ^e^DASS = Depression, Anxiety and Stress Scale.
